# Venetoclax plus hypomethylating agents or low-dose cytarabine in acute myeloid leukemia: all that glitters is gold?

**DOI:** 10.1038/s41408-020-0281-x

**Published:** 2020-01-28

**Authors:** Felicetto Ferrara

**Affiliations:** Division of Hematology, Cardarelli Hospital, Naples, Italy

**Keywords:** Drug development, Haematological diseases

Dear Editor,

Currently, older patients with acute myeloid leukemia (AML) have more treatment options than ever before, either at diagnosis or relapse^1,2^. In particular, impressive results have been reported following combinations of venetoclax (Ven) plus hypomethylating agents (HMA), and at a less extent, of Ven plus low-dose cytarabine (LDAC) in older and/or unfit AML patients^3,4^. In the pivotal study of Ven + HMA^3^, 37% of patients achieved complete remission (CR) and an additional 30% CR with incomplete hematologic recovery (CRi) with median OS of 17.5 months. Noticeably, ~50% of patients presented with poor-risk cytogenetics, and the median age was 74 years. While these results are clearly superior to HMA alone, CR rates seem to be also superior in comparison with intensive chemotherapy (ICT), independently from the risk group, and survival curves indicate sustained clinical benefit. The above data have generated great enthusiasm across the hematologic community, so that Ven/HMA is increasingly used in daily practice, and is expected to represent the new standard of care for unfit AML patients. Given the extremely favorable results observed in patients with NPM1 and IDH mutations, an intriguing question is whether Ven/HMA would replace ICT also in fit older patients with AML.

## Unanswered questions

While enthusiasm and excitation for these results seem to be fully justified, a number of questions still remain unresolved: (1) will data of the Dinardo trial be reproduced in the real world? (2) Are really unfit patients the ideal candidates to receive the combination? (3) Should all patients with unfavorable genetic findings at presentation, independently from age, receive Ven/HMA? (4) How to select between azacitidine (AZA) or decitabine (DEC) in combination with Ven? (5) Is Ven/HMA therapy an ideal bridge to allogeneic transplantation? In this article, I will attempt to address the above questions on the basis of currently available data.

## VEN plus HMA in the real world

Few data are currently available for Ven/HMA combination outside of clinical trials and even less in untreated patients. Winters et al. recently described the data from a small series of 26 patients unwilling or unfit for chemotherapy with a median age of 72 years^5^. CR/CRi were achieved in 73% of patients, CR in 46%. Of interest, four out of 14 evaluable patients (28%) obtained MRD negativity and none of them relapsed. Early death (within 60 days) occurred in 4 of 30 patients (13%), all due to disease progression. At a median of 113 days (9–394 days), there were 11 deaths after treatment, nine from disease progression, and two from infectious complications. Overall, the conclusion of the authors was that newly diagnosed AML patients treated in a “real-world” scenario had inferior outcomes compared with patients treated in the setting of a clinical trial. Zhang et al. compared 29 patients treated with Ven/HMA combination with 196 who received HMA monotherapy^6^. ORR as well as median time to response were more favorable for the combination in any risk group, while early mortality rate was not different. In conclusion, real-world data confirm the feasibility and the impressive response rate of Ven/HMA in high-risk as well as in high-risk older AML patients. Longer follow-up on a larger series of patients is clearly needed to draw any clinically relevant conclusion on survival.

## Are unfit patients the ideal candidates to receive the combination Ven/HMA?

In most studies focusing on patients defined as unfit, the majority of them were in PS 0–2; as an example, 83% of patients in Ven/AZA trial^3^ were in PS 0–1; therefore, it is not clear why most patients were considered as not eligible to ICT. On behalf of Italian Society of Hematology, we developed operative criteria, aimed at definition of patients not eligible to ICT or HMA, which have been validated in daily practice and largely adopted in Italy^7^. In my opinion, a major unresolved question is whether all patients who are candidates to HMA are also eligible to Ven/HMA, given that hematological toxicity and infectious risk is substantially higher for the combination. In our experience, overall toxicity is higher than single-agent AZA or DEC; therefore, it remains unclear whether Ven/HMA would be considered and given to really unfit patients. Finally, as shown in Table 1, different questions regarding inpatient or outpatient management, ideal antifungal prophylaxis, evaluation of response, and other uncertainties remain unanswered.

**Table 1:** Unanswered questions related to clinical management of AML patients with the combination Ven/HMA.

(1) Who would be given VEN/HMA?
a. Only > 65 years with adverse genetic and molecular findings?
b. All older patients with AML?
c. Transplant-eligible?
d. Young/adults within ELN-unfavorable risk group?
(2) How to define unfitness to VEN/HMA?
Are all patients selected for single-agent HMA eligible to Ven/HMA?
(3) How to give VEN/HMA?
a. In-/outpatient?
b. Ramp-up dose?
c. Which antifungal prophylaxis should be given?
d. Bone marrow aspirate/biopsy: after cycle 1? Cycle 2?
e. How many courses for definition of refractory ailments?
f. How to select between AZA and DEC?

## Should all older patients and young/adult patients with unfavorable genetic findings at presentation receive VEN/HMA?

Patients with high-risk AML, including unfavorable karyotype and tp53 mutations, are still incurable^8^. In particular, conventional chemotherapy in poor-risk older patients results in CR rate of <40% and anecdotal long-term survival^9^. The CR/CRi rate in the VEN/HMA trial was 60% in the group with adverse karyotype and 47% in tp53-positive patients^3^; therefore, in spite of the limited number of patients, it appears quite appropriate to administer Ven/HMA above the age of 60/65 years in this setting. Notwithstanding, the CR rate of 83.7% for pts with IDH1/IDH2 mutations, 84.6% for pts with NPM1 mutations, 59.5% for pts with tP53 mutations, and 53.3% for pts with FLT3 mutations were reported^10^, raising the question about the potential inclusion of all older patients with AML in VEN/HMA. Up to now, there is no definitive answer to this question, and prospective randomized trials are needed.

The therapeutic scenario is even more complex in young adult patients with high-risk disease in whom, following ICT, CR approximates 50% with consistent possibility of receiving allo-SCT, which represents the only curative option. As assessed at ClinicalTrials.gov website, only two trials are recruiting young adults with untreated AML: the first at the University of Chicago including tp53 mutant AML and adverse risk cytogenetics including any of the following: 3 or more abnormalities; deletions involving chromosomes 5, 7, or 17; abnormalities in chromosome 11 involving MLL; t(6;9); inv^3^ or t(3;3); the second at the University of Colorado, which enrolls subjects with non-APL and non-core-binding factor AML by WHO criteria. Waiting for the results of these and future studies, we continue to consider ICT for young adult patients, independently from the ELN risk category.

## How to select AZA or DEC in combination with Ven?

In AML, experimental data demonstrate the shared mechanisms of action of AZA and DAC on DNA-mediated markers of activity, but distinctly different effects in their actions on cell viability, protein synthesis, cell cycle, and gene expression^11^. Up to now, there are no direct head-to-head data available to make objective comparisons between AZA or DEC, and perhaps, never we will have. In general, Dec is perceived as more cytotoxic; therefore, we prefer it in proliferative disease on the basis of leukocytosis > 10 × 10^9^/l at diagnosis, and we adopt identical criteria for the combination with Ven.

## Is Ven/HMA therapy an ideal bridge to allogeneic transplantation?

The upper age limit for eligibility to allo-SCT is continuously increasing, and the procedure is currently offered up to 75 years, and definitive evidence has been provided that single-agent HMA can represent a useful bridge to transplantation in either MDS or AML^11,12^. In the pivotal study by Dinardo et al., a total of 21 patients out of 145 (14%) discontinued from study to receive stem cell transplantation. No details are provided regarding the criteria for identification of the best candidates to allo-SCT; however, it is conceivable that patients were in CR and had good performance status (PS). In our experience, not eligibile to ICT in most cases corresponds to not eligibile to allo-SCT. However, in selected cases unfitness may be strictly dependent on AML-related complications; therefore, it is conceivable that, following adequate supportive care and specific treatment, PS can substantially improve, namely in patients achieving CR or CRi. Obviously, higher CR/CRi rates following Ven/HMA therapy are expected to result in a higher percentage of patients who become eligible to allo-SCT.

## Conclusions

The proverb “all that glitters is not gold”, is stated to have been first used by William Shakespeare in his famous play, The Merchant of Venice, published in 1595, and means that something may not be as beneficial or as valuable as it appears.

Exciting results from Ven/HMA and Ven/LDAC led FDA to accelerated and continued approval for AML patients aged over 75 years and/or unfit for ICT. However, this indication may be contingent upon verification and description of clinical benefit in the confirmatory trial. The phase 3 studies, VIALE-A and VIALE-C, which are evaluating Ven in combination with AZA or LDAC versus single-agent Aza or LDAC, respectively, with overall survival as the primary endpoint, are intended as the confirmatory trials. In addition, data from real world are expected in order to confirm safety and efficacy. Finally, different issues concerning optimization of treatment, universally accepted criteria for timing and evaluation of response, and role in poor-risk young adults as well as older fit patients remain to be definitively clarified^13,14^. Further investigations in order to evaluate the opportunity and optimal strategy of specific antimicrobial prophylaxis agents in patients treated with Ven/HMA are necessary for clarity on this issue^15^. In Table 2, the pros and cons of VEN/HMA and ICT are summarized.

**Table 2:** Pros and cons for Ven/HMA and ICT for older AML-fit patients.

	Pros	Cons
Ven/HMA	Possible outpatient management Low early mortality rate High response rate in either intermediate or unfavorable ELN risk categories	Undefined duration of therapy Complex antifungal prophylaxis Uncertainty on response evaluation Poor outcome at progression/relapse
ICT	Short-term therapy Fast bridge to allo-SCT	Low response rate in poor-risk patients Prolonged hospitalization Potentially high early mortality rate Toxicity restricting eligibility to allo-SCT
